# Drivers and facilitators of the illegal killing of elephants across 64 African sites

**DOI:** 10.1098/rspb.2022.2270

**Published:** 2023-01-11

**Authors:** Timothy Kuiper, Res Altwegg, Colin Beale, Thea Carroll, Holly T. Dublin, Severin Hauenstein, Mrigesh Kshatriya, Carl Schwarz, Chris R. Thouless, Andrew Royle, E. J. Milner-Gulland

**Affiliations:** ^1^ Centre for Statistics in Ecology, Environment and Conservation, Department of Statistical Sciences, University of Cape Town, Rondebosch 7700, South Africa; ^2^ Interdisciplinary Centre for Conservation Science, Department of Zoology, University of Oxford, 11a Mansfield Road, Oxford OX1 3SZ, UK; ^3^ Department of Biology, University of York, York, UK; ^4^ UN programme for Monitoring the Illegal Killing of Elephants, Nairobi, Kenya; ^5^ Technical Advisory Group to the programme for Monitoring the Illegal Killing of Elephants, University of Freiburg, Germany; ^6^ Department of Biometry and Environmental System Analysis, University of Freiburg, Germany; ^7^ StatMathComp Consulting, Port Moody, British Columbia, Canada; ^8^ Save the Elephants, Nairobi, Kenya; ^9^ U.S. Geological Survey, Eastern Ecological Science Center, MD, USA

**Keywords:** Monitoring the Illegal Killing of Elephants, illegal wildlife trade, wildlife poaching, corruption, poverty, wildlife crime

## Abstract

Ivory poaching continues to threaten African elephants. We (1) used criminology theory and literature evidence to generate hypotheses about factors that may drive, facilitate or motivate poaching, (2) identified datasets representing these factors, and (3) tested those factors with strong hypotheses and sufficient data quality for empirical associations with poaching. We advance on previous analyses of correlates of elephant poaching by using additional poaching data and leveraging new datasets for previously untested explanatory variables. Using data on 10 286 illegally killed elephants detected at 64 sites in 30 African countries (2002–2020), we found strong evidence to support the hypotheses that the illegal killing of elephants is associated with poor national governance, low law enforcement capacity, low household wealth and health, and global elephant ivory prices. Forest elephant populations suffered higher rates of illegal killing than savannah elephants. We found only weak evidence that armed conflicts may increase the illegal killing of elephants, and no evidence for effects of site accessibility, vegetation density, elephant population density, precipitation or site area. Results suggest that addressing wider systemic challenges of human development, corruption and consumer demand would help reduce poaching, corroborating broader work highlighting these more ultimate drivers of the global illegal wildlife trade.

## 1. Introduction

The illegal wildlife trade is one of the most high-value illicit trade sectors globally, threatening both human well-being and biodiversity [1,2]. African elephant populations have experienced significant declines (approx. 30%) since 2006 [3,4], correlating with high rates of illegal killing [5,6] and large seizures of trafficked ivory [7,8]. This threat to a charismatic species results in lost tourism revenues for African states [9], dilutes the important ecosystem function of elephants [10] and results in both hunters and rangers losing their lives [11,12]. Conservation responses have involved a diversity of local and international interventions, from law enforcement and community engagement at the local level, to demand reduction and global ivory trade bans.

**Figure 1.**
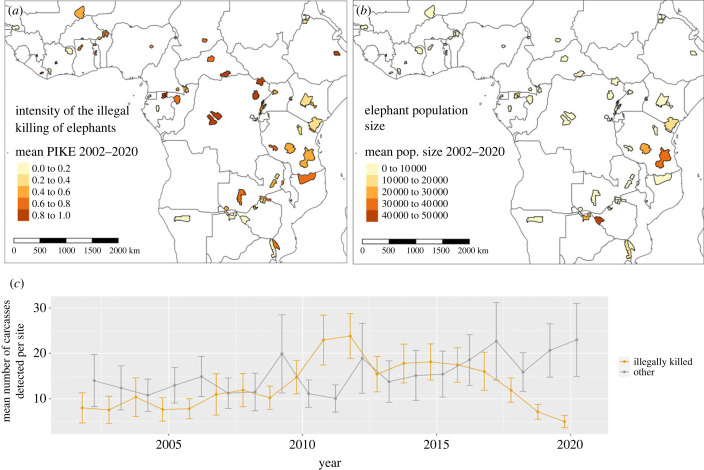
The 64 African sites contributing to the programme for Monitoring the Illegal Killing of Elephants (MIKE). (*a*) The intensity of the illegal killing of elephants at each site, measured as the Proportion of Illegally Killed Elephants (PIKE; see Methods). (*b*) Mean elephant population sizes from the African Elephant Database (4). (*c*) The mean number of carcasses detected per site (mostly by wildlife rangers) between 2002 and 2020.

Our aim in this research was to help inform strategies to tackle elephant poaching by empirically identifying local to global factors that may drive or facilitate poaching across Africa. The Convention on the International Trade in Endangered Species of Fauna and Flora (CITES) established the Monitoring of the Illegal Killing of Elephants (MIKE) programme in 2002 to monitor rates of illegal elephant killing at over 90 sites in Africa and Asia [13] (figure 1). MIKE monitors poaching levels and trends by analysing data associated with elephant carcasses detected at MIKE sites. According to MIKE protocols, illegal killing includes poaching to harvest ivory as well as mortality related to human-elephant conflict (though only approximately 3% of all carcass records are associated with conflict [14]). Trends in illegal killing from multiple sites are aggregated to the sub-regional and continental levels to help inform international decisions on the ivory trade and elephant conservation at various inter-governmental wildlife trade forums [13]. The intensity of illegal killing for each site and year is measured as the Proportion of Illegally Killed Elephants (PIKE; see Methods). By using PIKE as an index of relative poaching rates and by considering patterns across all populations, we seek to identify general drivers/facilitators of illegal killing across the continent. Our analysis does not, therefore, necessarily identify factors that may be important at a few sites where absolute numbers of illegally killed elephants may be high.

When seeking to identify factors associated with elephant poaching, it is essential to understand what drives the decisions of key actors in the system. It is important to explore factors that may help explain the full range of drivers and facilitators of illegal killing. Oyanedel *et al*. [15] review two main approaches to studying crime and non-compliance with rules: the actor-based approach considers the motivations of individual people to comply or not, while opportunity-based approaches consider how the immediate environment/context may create opportunities for non-compliance. For example, poverty may act on the motivations of individuals to be complicit in illegal killing, while corrupt park officials or low law enforcement capacity may create the context that facilitates this killing. Poaching of high-value species like elephants and rhinoceroses is driven primarily by criminal networks or syndicates as opposed to opportunistic subsistence hunters [7,16–18]. Why do these networks choose to operate in the countries and sites that they do, at the times and in the ways that they do? A second set of decision-makers are individuals who choose to join hunting operations on the ground, to be complicit with, or turn a blind eye to, illegal killing in their local areas. The connection between higher-level syndicates and local poachers is often fluid, with syndicates relying on middlemen to acquire ivory from a wide array of poachers [19]. We are interested in understanding what factors influence the decisions of both groups.

To address our research aim, we took a hypothesis-driven approach that involved four stages:

(1) First, we reviewed evidence from the literature to generate hypotheses about socio-economic, political and environmental factors (or covariates) that may plausibly drive, facilitate, motivate or hinder the illegal killing of elephants at different scales (from site-level to national to global).
(2) Second, for each covariate identified we reviewed available datasets and assessed how well they represented the factor of interest (for example, we assessed four alternative measures of wealth/poverty).
(3) Third, we ranked each covariate by both the plausibility of the hypotheses associated with it (strength of logic and evidence in the literature) and the quality of available datasets.
(4) Fourth, covariates with adequately high plausibility and data quality were tested for associations with annual data on the illegal killing of elephants from 64 African MIKE sites in 30 countries over 19 years (2002–2020; figure 1). This established the degree of support for each hypothesis in (1). We fitted a Bayesian hierarchical generalized linear mixed model (GLMM) to the poaching/covariate data, with site, year, site-year, and country random effects to fit the data structure. Model selection was performed using LASSO regularization. Regularization and multiple random effects tend to reduce the effect sizes and precision of poorly supported covariates [20], helping ensure that only those covariates with strong empirical associations with the illegal killing of elephants were identified as important (see Methods).

We build on similar previous analyses of correlates of elephant poaching [21,22] by taking advantage of several years of additional poaching data, data from several additional sites, as well as improved covariate datasets not previously tested (table 1). This includes geo-referenced data on armed conflicts in the vicinity of monitored elephant populations [23], internationally comparable wealth and development data recently constructed from long-term surveys of households adjacent to monitored sites [24,25], improved measures of site-level law enforcement capacity (updated MIKE assessments; see electronic supplementary material S2), data on site accessibility [26], and a newly collated global dataset on 3012 raw elephant ivory price samples [27] as a proxy for ivory demand (table 1). Furthermore, our extensive review of evidence to generate and interrogate specific hypotheses and associated data sources further advances previous work and helps us better scrutinize possible mechanisms underlying complex relationships, such as those between illegal killing and poverty or armed conflict.

**Table 1.** The 12 factors/covariates (out of 20 reviewed) identified as having sufficient plausibility and data quality for testing for empirical associations with the illegal killing of elephants (PIKE; Proportion of Illegally Killed Elephants). Evidence for the hypothesis underlying each covariate, the candidate data sources reviewed for each covariate (e.g. four measures of wealth/poverty were considered), details on how data were extracted to sites/years/countries, and information on the eight excluded covariates are included in electronic supplementary materials S2 and S3. All correlations between covariates were rless than 0.6, except wealth and development which were modelled separately (see Methods and electronic supplementary material, figure S1).

factor (plus proxy data and link)	hypothesis for how factor might influence poaching (PIKE)	scale
drivers: factors hypothesized to drive illegal killing		
ivory demand (annual trend in global elephant ivory price)	ivory demand may incentivize illegal killing; if demand increases (e.g. due to increased disposable income) and supply cannot meet demand, ivory price may increase and further incentivise illegal killing^a^	global-by-year
facilitators: factors hypothesised to facilitate illegal killing and ivory trafficking		
governance quality (World Governance Indicators)	poor governance may facilitate illegal killing at the site level and the trafficking of ivory within and out of source countries as officials (park managers and border staff) accept bribes or turn a blind eye	country-by-year
accessibility (travel time from site to the nearest city)	sites that are easier for syndicates and hunters to access, and from which ivory can be easily and quickly transported, may experience higher levels of illegal killing	site
accessibility (size/area of site)	smaller sites have a higher edge/area ratio making it easier for hunters to access and leave quickly, while larger sites may be difficult to police	site
armed conflict (Total battle deaths per site-year derived from the *Uppsala* Conflict Geo Dataset)	armed conflicts lead to institutional and socioeconomic changes that may facilitate illegal killing, or ivory may be used to fund the operations of warring militias	site-by-year
elephant populations (size and density)	sites with larger or more dense elephant populations may be more attractive targets to hunters and syndicates due to higher encounter rates	site-by-year
motivators: factors hypothesized to increase or decrease the motivation to poach elephants		
household wealth (sub-national household wealth)	the socio-economic conditions of poverty may compel individuals to engage with illegal killing to earn income to meet basic needs, in the absence of viable alternatives	site-by-year
human development (sub-national human development index - income/health/education)	less developed communities (not necessarily in poverty) may be more likely to participate in or facilitate illegal killing to earn extra income or through turning a blind eye	site-by-year
law enforcement capacity (MIKE LE Capacity Assessments)	enhanced law enforcement allows for more committed and effective rangers, more effective apprehension and deterrence, and may thus result in lower illegal killing	site
others: confounding factors which are unrelated to illegal killing but that may influence the PIKE index		
precipitation/drought (rainfall anomaly from CHIRPS data)	PIKE is sensitive to natural mortality rates, so factors explaining natural mortality variation (e.g. rainfall/drought) may explain variation in PIKE both among sites and over time within a site	site-by-year
carcass detectability (vegetation density from MODIS NDVI)	densely vegetated sites may have higher PIKE due to low detectability of natural mortalities which do not have the same detection cues as illegally killed carcasses (forest may also help conceal hunters)	site-by-year
elephant species (forest or savannah) (delineation from IUCN Red List assessments)	for various difficult to measure reasons, previous evidence suggests forest elephants may suffer higher poaching rates than savannah elephants, which may explain variation in the PIKE index across the continent	site (population)

^a^We identified price as the best demand proxy, though price is dynamically determined by both supply and demand (see electronic supplementary material S2 for a full discussion).

## 2. Methods

### (a) MIKE sites and data on the illegal killing of elephants

Here we use 19 years (2002–2020) of annual elephant carcass data (collected mostly by wildlife rangers) from 64 protected sites in 30 African countries (figure 1). Levels of illegal killing are estimated for each site, each year, as PIKE: the number of illegally killed elephant carcasses detected as a proportion of all carcasses detected (including natural mortalities, management-related deaths, and mortalities of unknown cause). Some sites were established more recently, and each site has a variable number of years of PIKE data (figure 1), so our final dataset consisted of 780 site-year observations of PIKE. The PIKE index is subject to several biases (such as sensitivity to natural mortality variation and higher detectability of poached versus natural mortalities in different habitats), but also has several advantages such as being relatively robust to variation in patrol effort and elephant density (see https://citesmike.org/analysis for a full discussion). The index has also been profitably used in various published analyses [6,21,22]. Our rainfall anomaly covariate also partly controls for changes in drought-related natural mortality (table 1).

### (b) Statistical model

To match the data structure, we used a Bayesian hierarchical GLMM with a binomial error structure to determine which covariates had a strong empirical association with PIKE across sites, countries and years. We used a PIKE-covariate model previously developed by Hauenstein *et al*. [22] with the significant addition of a site-year random effect alongside the site, country and year random effects. This error structure was chosen to represent the data structure, account for pseudo-replication at the different levels, and ensure a more conservative interpretation of main effects. The site-year effect deals with pseudo-replication of multiple carcass observations within a site-year while also reducing the possibility of false positives for the main site-year effects like wealth and armed conflict (by reducing effect precision, [20]). The site-year effect also substantially improved model fit (Bayesian *p*-value test for goodness of fit; see below). Model selection was performed using LASSO regularization which penalizes overly complex models by shrinking covariate effects toward zero [28]. Our model was conservative in that the multiple random effects and LASSO regularization ensured that a very strong empirical association between a particular covariate and PIKE is required for sufficient evidence of an effect.

We model PIKE for each site-year observation by treating the number of illegally killed carcasses detected ($N.\mathrm{illega}l_{sy}$) at each site (*s*) and year (*y*) as a binomial random variable:

$$N.\mathrm{illega}l_{sy}\;\tilde{\;}\mathrm{Binomial}\;({\mathrm{PIKE}}_{sy},N.\mathrm{tota}l_{sy}),$$

where $N.\mathrm{tota}l_{sy}\;$is the total number of carcasses detected at each site and year. We then model PIKE as a function of the 11 covariates and normally distributed random intercepts (N) for site, site-year, year, and country

$$\begin{array}{rl} \mathrm{logit}({\mathrm{PIKE}}_{sy}) & =\beta_{0}+\;\overset{6}{\underset{k=1}{\sum }}\beta_{k}X_{sy}+\;\beta_{7}{\mathrm{Gov}}_{\mathrm{country}\;∋\;s,\;y}+N(\mu_{\mathrm{site}},\sigma_{\mathrm{site}})\\ & \;+N(\mu_{\mathrm{year}},\sigma_{\mathrm{year}})+\;\;N(0,\sigma_{\mathrm{site}-\mathrm{year}})+N(0,\sigma_{\mathrm{country}}) \end{array}$$

Where ${\mathrm{Gov}}_{\mathrm{country}\;∋\;s,\;y}\;$represents the governance quality of the country that contains site *s*, in the year *y*. $\;X_{sy}$ represents the six site-by-year covariates (table 1). We model the hierarchical level means for the site random intercept ($\mu_{\mathrm{site}}$) as a function of the site covariates that had only one measurement across all years (area of site, law enforcement capacity, and travel time to the nearest city):

$$\mu_{\mathrm{site}}=\beta_{9}{\mathrm{Area}}_{\mathrm{site}}+\;\beta_{10}{\mathrm{LawEnf}}_{\mathrm{site}}+\;\beta_{11}{\mathrm{TravelTime}}_{\mathrm{site}}.$$

Finally, we model the hierarchical level mean for the year random intercept as a function of the global trend in the price of elephant ivory:

$$\mu_{\mathrm{year}}=\beta_{12}{\mathrm{IvoryPrice}}_{\mathrm{year}}.$$

We fitted the model using Markov chain Monte Carlo (MCMC) sampling, implemented using the software JAGS [29], integrated with the R package R2jags [30]. We found that 100 000 MCMC iterations with a 50 000 burn in was sufficient to ensure convergence, which was confirmed by visual examination of chain-iteration trace plots as well as Gelman Rubin potential scale reduction factor ($\hat{R}$) values of less than 1.1. We used gamma (1,1) priors for the standard deviations of the site, year, site-year and country random intercepts, and Laplace priors on the covariate coefficients to achieve LASSO regularization (see [22] for details). All covariates were Z-transformed to ensure the same scale.

To test model fit, we used the model equation to simulate response (PIKE) data and then compared discrepancy measures (observed versus predicted) for both the empirical and simulated data using Bayesian *p*-values [31]. Most covariates had complete data, however, the trend in ivory price was missing data for the years 2016–2020, rainfall anomaly data were missing for the year 2020, governance data were not available for 2020, and law enforcement capacity and community participation data were missing for 6 of the 64 sites. We imputed missing data for these covariates using draws from a standard normal distribution, noting that covariates were standardized to this scale [32]. Finally, the ‘shrinkage’ effect helped ensure that estimated covariate effects were influenced more by sites with more reliable estimates of PIKE (by virtue of larger carcass sample sizes) [21].

To test model predictive performance, we split the raw data into training and testing sets, using a 75% to 25% random split. We then compared observed PIKE values to median PIKE estimates for the testing set, based on 5000 MCMC samples of the model fitted to the training data only, and calculated R^2^ values for the correlation. Then, to account for spatial dependencies in the data [33], we tested predictive performance by excluding 15 randomly selected MIKE sites (approx. 25% of all observations) for the training set and then followed the same procedures as above for testing. Finally, to estimate the proportion of spatial, temporal, and spatio-temporal variation in PIKE accounted for by the covariates (fixed effects), we compared the size of the variance components of the random effects in the full model to a model with only the random effects (a proportional change in variance analysis following equation 31 in [34]).

Due to correlations between the wealth and development covariates (electronic supplementary material, figure S1), we constructed several supplementary models for these covariates (see Results). Also, the literature suggests that the effects of armed conflict (disruptions to law enforcement, socio-economic change, corruption, and lawlessness) may not be immediate [17,35]. Therefore, we present models with conflict intensity measured for each site as the total battle deaths in the current year, over 2 years (the current year and previous year), 3 years (the current and previous two years) and 5 years (the current and previous four years).

## 3. Results

We identified 20 plausible covariates of the illegal killing of elephants, of which a final set of 12 covariates (those with adequately high plausibility and data quality) were tested in the statistical model to establish support for the hypotheses underlying their influence on the illegal killing of elephants (table 1). More detail on how each covariate considered in our analysis may relate to the decision-making of criminal syndicates is included in electronic supplementary material S2.

**Figure 2.**
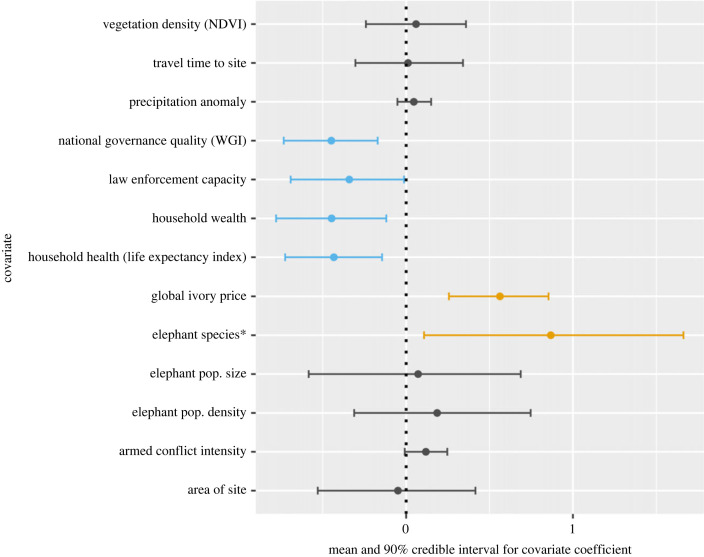
The effect of tested covariates on the illegal killing of elephants (PIKE), based on the LASSO-regulated Bayesian GLMM. Blue lines (coefficient values less than 0) represent covariates with strong evidence for a negative effect (illegal killing tends to decrease as the covariate increases), while orange represents a strong positive effect. Points and bars represent mean and 90% credible intervals for covariate coefficients (5000 MCMC posterior samples). Covariates were standardized so coefficient effect sizes are directly comparable. Elephant species was coded as 0 for sites with savannah elephant (*Loxodonta africana*) populations and 1 for those with forest elephant populations, so values greater than 0 represent higher estimated illegal killing for forest elephants.

**Figure 3.**
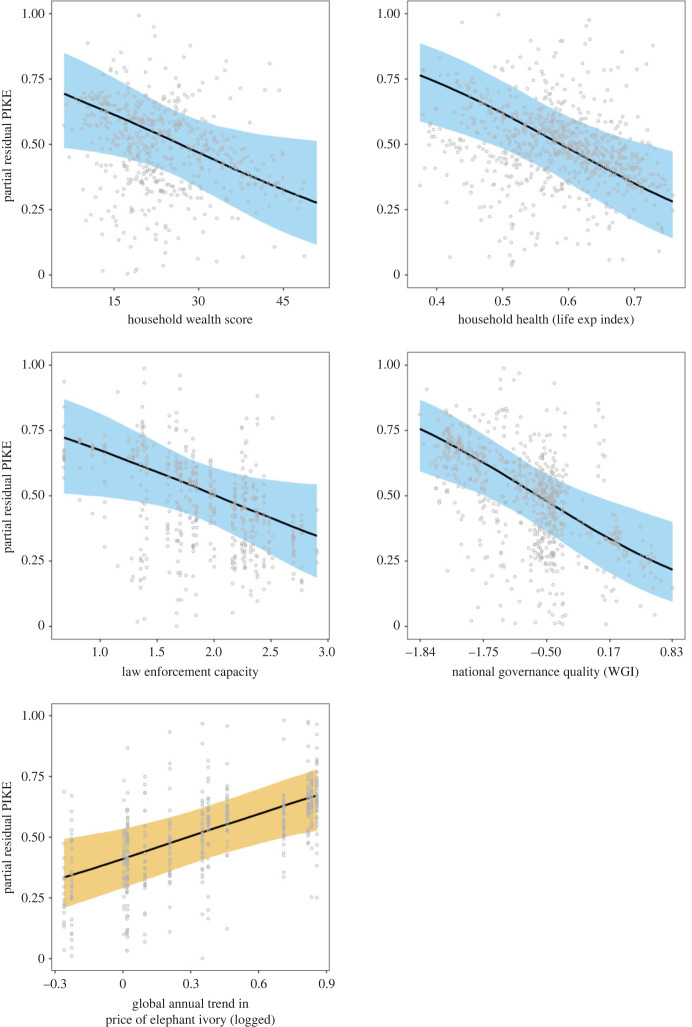
The estimated effect of well-supported covariates (90% credible interval for their effect excludes zero) on the proportion of illegally killed elephants (PIKE), as represented by conditional effects (partial residuals account for other covariates and random effects) from the LASSO-regularized Bayesian GLMM. Bands represent 90% credible intervals from 5000 MCMC samples, and grey circles represent response-scale partial residuals. Orange = positive association, blue = negative associations. The units for ivory price represent median residuals from a regression of log-transformed price data against several control variables (see [9]).

We found evidence for negative associations between the illegal killing of elephants and each of national governance quality, site-level law enforcement capacity, and the wealth and health of households in the vicinity of MIKE sites (Bayesian GLMM 90% credible intervals for covariate coefficients do not include zero: figures 2 and 3). The credible interval for armed conflict intensity suggests that sites with more intense conflict (higher total battle deaths by site and year) tend to have higher rates of illegal killing, but the evidence is not strong (figure 2, 90% credible interval includes zero). We find no evidence for effects on the illegal killing of elephants of the precipitation anomaly, vegetation density, elephant population size and density, travel time from the site to the nearest city or site area (km^2^). We also found evidence for a positive association between the global annual trend in the price of elephant ivory (based on 3012 raw ivory price samples; see electronic supplementary material S2) and the temporal trend in the illegal killing of elephants as represented by PIKE (figure 2). Finally, we found evidence that forest elephant populations tended to suffer higher rates of illegal killing than savannah elephant populations (figure 2).

**Figure 4.**
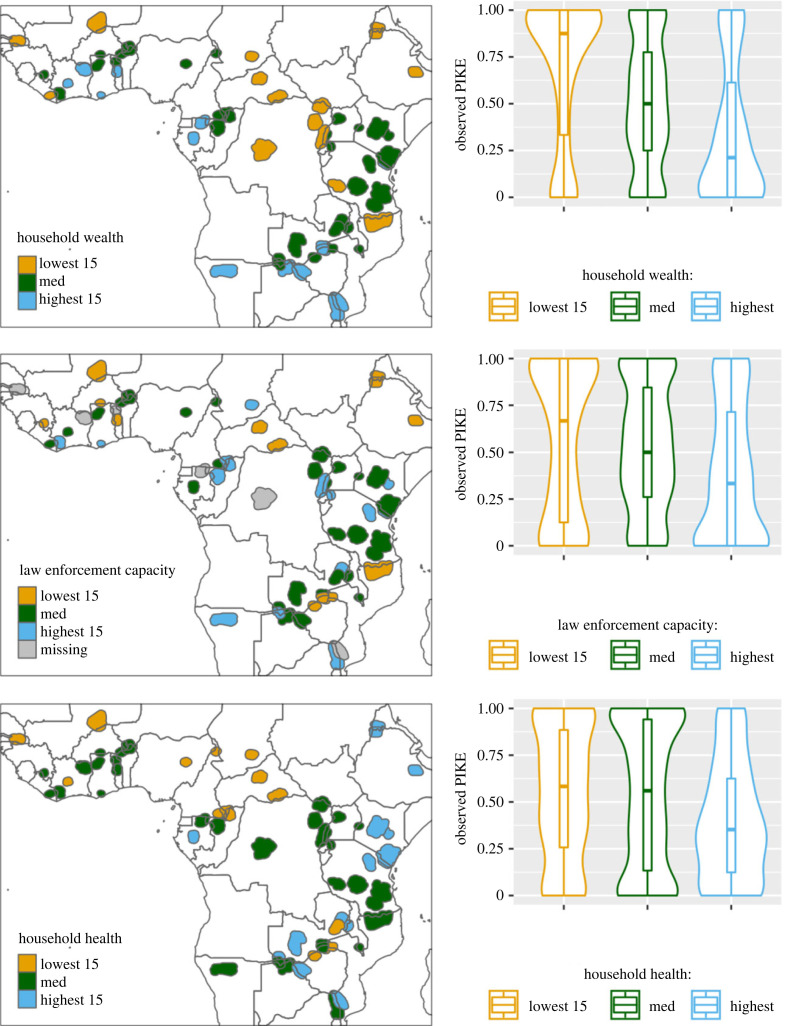
Right panels: observed PIKE (Proportion of Illegally Killed Elephants) for different categories of MIKE sites ordered by the well-supported site-level covariates (household wealth, household health and law enforcement capacity), with categories representing the 15 MIKE sites with the highest and lowest mean values for each covariate. ‘Med’ represents the 34 sites with intermediate values for each covariate (there were 64 sites with data in our sample). Observed PIKE is summarized using violin plots (showing data distribution kernels) and box plots (horizontal lines are median and upper/lower quartiles). Left panels: maps of the location of the MIKE sites in each of the categories for each covariate.

We also found a strong negative association between human development and the illegal killing of elephants (electronic supplementary material, figure S2), a strong negative association between the illegal killing of elephants and the health and income dimensions of subnational human development, and a positive association between illegal killing and the education dimension (see electronic supplementary material, figure S3). Focusing in on the best-supported site-level covariates, there are relatively consistent geographical patterns in the location of the top and bottom 15 sites for household wealth and health, but variation in law enforcement capacity is spread across the continent (figure 4).

We explored the effect of armed conflict intensity further, to test whether the time-period over which it is measured affects associations. Conflict aggregated over the current and previous year had a strong positive association with the illegal killing of elephants (90% credible interval excludes zero: electronic supplementary material, figure S4). However, we found a weaker association with conflict in the current year alone, and no evidence for an effect of conflict intensity when measured over three- and five-year periods (electronic supplementary material, figure S4).

Model predictive capacity was adequate to high, with *R^2^* of 0.36 for prediction of PIKE at 15 excluded sites (90% CI 0.07–0.51) and *R^2^* of 0.73 (90% CI 0.62–0.81) for a random test-train split (see Methods). Bayesian *p*-values greater than 0.40 (see Methods) confirmed model fit for the main and supplementary models, as did a plot of observed versus predicted PIKE values (electronic supplementary material, figure S5). The variance components of the site, year, site-year, and country random effects in a random-effects-only model did not reduce significantly when the covariates were added in the full model (electronic supplementary material, figure S6). This indicates that the covariates left a large amount of unexplained variation in the illegal killing of elephants, though covariates were better at explaining spatial versus temporal variation (larger declines in the variance components for the spatial random effects; electronic supplementary material, figure S6). The lambda parameter for the LASSO regularization in the main model was large (1.92, 90% credible interval: 1.26–2.64) indicating relatively high shrinkage of covariate effects toward zero (suggesting strong evidence for observed covariate effects).

## 4. Discussion

The unsustainable and illegal killing of elephants for ivory is ongoing across Africa [5,6]. We found evidence to support the hypotheses that strong national governance, higher levels of local human development (health and wealth), and stronger site-level law enforcement capacity help mitigate elephant poaching. We also found evidence consistent with the hypothesis that demand-driven increases in ivory price may lead to greater incentives for illegal killing of elephants across Africa. Addressing these systemic drivers of poaching will require wider policies and interventions beyond the traditional remit of biodiversity conservation, such demand reduction in consumer countries, reforms to government institutions to promote greater accountability and transparency, and programmes to promote adequate access to educational, health and economic opportunities where they are lacking. While such interventions are of course an enormous task and already at the forefront of the global Sustainable Development Goals, our results suggest they will have co-benefits for biodiversity conservation.

Hauenstein *et al*. [22] found similar tosociations between Africa-wide elephant killing and poverty, corruption, and ivory price. However, we used a more direct and finer-scale measure of poverty (household wealth, rather than infant mortality rate), a more direct measure of ivory prices (global elephant ivory price compared to mammoth ivory prices), and data for 11 additional sites and three additional years. We also used a more comprehensive measure of law enforcement capacity than Hauenstein *et al*. (see electronic supplementary material S2) and found stronger evidence for a mitigating effect on illegal killing. In another similar analysis, Schlossberg *et al*. [36] did not find correlations between elephant mortality and human national human development or governance, although they acknowledge lower statistical power (they focused on savannah elephants in 17 countries while we focus on both savannah and forest elephants across 30 countries). We considered using Schlossberg *et al*'s [36] measure of poverty (the Night Lights Poverty Index), but most MIKE sites are in rural areas so there is little contrast in light intensity among sites. Our household wealth dataset is based on a local, direct, and internationally comparable metric of material well-being [24] and had greater contrast among sites.

The health dimension of subnational human development that we used is based on the under-5 mortality rate [25], so the observed positive association with PIKE accords with Hauenstein *et al*. [22] who found that PIKE was positively associated with infant mortality rate. However, our household wealth and health (infant mortality) covariates were not strongly correlated, and both had an effect, suggesting that wealth levels affect poaching over and above the health effects observed here and by [22]. Thus, our results provide more conclusive evidence that illegal elephant killing is related to local poverty.

Our observed wealth effect provides support for the hypothesis that local socio-economic deprivation may increase the likelihood of elephants being illegally killed. One interpretation might be that in areas of economic deprivation, local residents participate in illegal killing to meet their basic needs or earn extra income, in the absence of viable alternatives. Another interpretation might be that criminal ivory syndicates seeking to recruit local hunters target these areas because they are able to operate more effectively there (for a range of possible reasons). Previous work points to exceedingly high levels of illegal killing in central Africa and the northern Mozambique southern Tanzania landscape [8,37], which may explain our results, in that MIKE sites in these regions had among the lowest household wealth scores (figure 4). Wealth scores near all MIKE sites were low by international standards (less than 45 on a 0–100 scale [24]), yet we still found that PIKE was higher for areas in more extreme poverty. This contrasts with previous ethnographic work suggesting that individuals involved in illegal killing of high-value species like rhinoceros and elephant are often not in poverty [38,39]. The positive association between illegal killing and the education dimension of the subnational Human Development Index accords with some anecdotal evidence from the Serengeti and Katavi ecosystems in Tanzania where poachers were found to be generally well-educated (which may facilitate selection by syndicates). However, causal hypotheses need deep understanding through more focused site-level research before they are accepted as the reason behind observed associations [40].

Market demand for wildlife products is one of the most well-evidenced factors driving the global illegal wildlife trade [1,41]. The positive ivory price effect we observed supports the hypothesis that demand-driven increases in ivory price may lead to greater incentives for illegal killing, and accords with previous work [5,22]. While price is not a direct measure of demand, and there may be multiple mechanisms behind a positive price-PIKE relationship, we considered price to be the most robust available proxy for ivory demand (see electronic supplementary material S2 for a full discussion). However, the relationship between ivory price and illegal killing may be reciprocal (price affects motivations to supply ivory and supply affects price) and stockpiling and speculative trading in ivory are known to occur. Notably, a comprehensive recent analysis by Do *et al*. [27] of associations between a proxy (instrumental variable) for ivory price and PIKE found an inelastic relationship, whereby PIKE increased less-than-proportionately as price increased [27]*.* They used gold price as their instrumental variable to control for possible endogeneity (whereby ivory price is correlated with other unmeasured drivers of illegal killing). However, low elasticity between price and PIKE does not necessarily imply no relationship, but rather that the effect is small in the observed data range. Given the close correlation Do *et al*. [27] found between ivory and gold prices, it is possible that our positive ivory price effect may be due to geopolitical shifts in the global economy (as also reflected in gold prices) rather than factors specific to the ivory market.

Our results provide support for the hypothesis that enhanced law enforcement capacity reduces the illegal killing of elephants (which may operate through apprehension or deterrence of offenders). Criminal syndicates are more likely to target areas where the risk of apprehension is lower [15]. Similar evidence was found in studies in Tanzania, Zambia and Malawi [42–44]. Although we selected our law enforcement covariate as the most robust of several considered (electronic supplementary material S2), it does not account for changes in law enforcement capacity over time and the tendency to under- or overestimate law enforcement capacity may vary by site according to personnel (although experienced personnel provided the assessments). Finally, it is also possible that sites with higher law enforcement and patrolling capacity detect a higher proportion of available natural mortalities, which would lead to lower PIKE scores.

The link between corruption and organized is well established in the literature [45]. There is growing evidence that poor governance may negatively affect various aspects of biodiversity protection [46–48]. We observed governance quality to be strongly and negatively associated with the illegal killing of elephants, as in previous analyses of similar data [21,22]. Our result also accords with Bennett [49] who describes how bribery and corruption opportunities exist all along ivory supply and value chains, where officials may turn a blind eye to, or actively engage in, site-level illegal killing and ivory trafficking within and between countries. van Uhm & Moreto [50] found that wildlife poachers in Uganda, Russia, China and Morocco and traders may interact with government enforcement agents in a diversity of corrupt ways that can facilitate harvest, transport, processing and export of wildlife products.

The strong elephant species effect suggests that forest elephants on average suffer higher rates of illegal killing compared to savannah elephants [5,37]. The species effect is interesting in that it is over and above any effect due to differences between savannah and forest elephant populations in vegetation density, precipitation, population density, or any other effects already captured in other covariates. This may, however, represent a geographical region effect as the vast majority of forest elephant populations are in West and Central Africa while savannah populations mostly occupy East and Southern Africa. One possible explanation might be that natural mortalities tend to be harder to detect in forested environments, artificially inflating PIKE estimates. However, we would expect the vegetation density covariate to capture this effect. Maisels *et al*. [37] highlight expanding infrastructure and encroachment into core elephant habitat as a key driver of forest elephant poaching. While the difference might be explained by demand for harder Forest elephant ivory for certain items such as name seals and musical instrument components in key consumer countries like Japan, this specific demand has largely declined since its peak in the 1970s and 1980s [51].

It is important to note that our analysis does not necessarily identify factors that have led to the largest absolute number illegally killed elephants. It is possible that the factors driving large numbers of elephant killings at a handful of sites (such as observed for Selous and Rungwa/Ruaha in Tanzania around 2010–2013) may be different from the drivers/facilitators of illegal killing that are general across sites (as identified in our analysis). However, the goal of this paper is to find common patterns across the continent, rather than try and explain drivers of poaching at a few key ‘hotspot’ sites. Furthermore, genetic seizure analyses suggest poaching hotspots have shifted over the last 20 years, the period of our analysis [8,52]. Our analysis across the whole continent and relatively long time period means we can learn something useful about tackling future hotspots.

Our results must be considered in the light of the limitations of the underlying datasets. MIKE data do not cover all Africa elephant populations and the PIKE index may be sensitive to natural mortality rates and differential detectability of illegally killed carcasses and natural mortalities (see Methods). Also, measuring factors like wealth and law enforcement accurately and in a comparable way over many sites and countries is difficult, and so covariate data may be biased and incomplete. Furthermore, many plausible drivers of the illegal killing of elephants cannot be adequately captured in a covariate. Global one-off events, or significant local events, may influence illegal killing but remain unmeasured. Finally, our analyses of proportional change in variance and model predictive performance suggests that much variation in PIKE remains unexplained by our covariates. This is perhaps not surprising given that illegal killing is influenced by a complexity of human decision-making within equally complex social and political institutions and networks affecting both offenders and law enforcement, which themselves interact with ecological factors and change over time. It is likely that there are many site, year, and country-level idiosyncrasies that cannot easily be captured in a covariate.

Notwithstanding these caveats, our approach of seeking a hypothesis-driven *a priori* understanding of the dynamics of illegal elephant killing and management, identifying the best available covariates to represent these dynamics, and using a tailored statistical modelling approach, helped us shed light on drivers and facilitators of illegal elephant killing across Africa. Overall, our results suggest that addressing system-level challenges at a variety of scales (poor governance, low human development and ivory market dynamics) is essential to tackling illegal elephant killing, alongside the traditional focus on law enforcement. This corroborates broader work that has highlighted the importance of these more ultimate drivers of the global illegal wildlife trade [1,40,53].

## Data Availability

Raw data, R statistical code and instructions for reproducing this analysis are available online within the Harvard Dataverse Repository: https://doi.org/10.7910/DVN/GNI6DS [54]. The data are provided in electronic supplementary material [55].

## References

[1] Sas-Rolfes M, Challender DWS, Hinsley A, Veríssimo D, Milner-Gulland EJ. 2019 Illegal wildlife trade: scale, processes, and governance. Annu. Rev. Environ. Resour. **44**, 201-228. (10.1146/annurev-environ-101718-033253)

[2] Esmail N et al. 2020 Emerging illegal wildlife trade issues: a global horizon scan. Conserv. Lett. **13**, 1-10. (10.1111/conl.12715)

[3] Chase MJ et al. 2016 Continent-wide survey reveals massive decline in African savannah elephants. PeerJ **4**, e234. (10.7717/peerj.2354) PMC5012305 27635327

[4] Thouless CR, Dublin HT, Blanc JJ, Skinner D, Daniel T. 2016 African elephant status report 2016: an update from the African elephant database. Gland, Swizerland: IUCN Species Survival Commission.

[5] Wittemyer G, Northrup JM, Blanc J, Douglas-Hamilton I, Omondi P, Burnham KP. 2014 Illegal killing for ivory drives global decline in African elephants. Proc. Natl Acad. Sci. USA **111**, 13 117-13 121. (10.1073/pnas.1403984111) PMC4246956 25136107

[6] Schlossberg S, Chase MJ, Gobush KS, Wasser SK, Lindsay K. 2020 State-space models reveal a continuing elephant poaching problem in most of Africa. Sci. Rep. **10**, 1-9. (10.1038/s41598-020-66906-w) 32576862 PMC7311459

[7] Underwood FM, Burn RW, Milliken T, Raul A, Montoya H. 2013 Dissecting the illegal ivory trade: an analysis of ivory seizures data. PLoS ONE **8**, e76639. (10.1371/journal.pone.0076639) 24250744 PMC3799824

[8] Wasser S, Brown L, Mailand C, Mondol S. 2015 Genetic assignment of large seizures of elephant ivory reveals Africa's major poaching hotspots. See http://science.sciencemag.org/content/349/6243/84.short (accessed 1 August 2016). 10.1126/science.aaa2457 PMC5535781 26089357

[9] Naidoo R, Fisher B, Manica A, Balmford A. 2016 Estimating economic losses to tourism in Africa from the illegal killing of elephants. Nat. Commun. **7**, 13379. (10.1038/ncomms13379) 27802262 PMC5097124

[10] Robson AS, Trimble MJ, Purdon A, Young-Overton KD, Pimm SL, van Aarde RJ. 2017 Savanna elephant numbers are only a quarter of their expected values. PLoS ONE **12**, e0175942. (10.1371/journal.pone.0175942) 28414784 PMC5393891

[11] Büscher B, Ramutsindela M. 2016 Green violence: rhino poaching and thewar to save southern africa's peace parks. Afr. Aff. **115**, 1-22.

[12] Belecky M, Singh R, Moreto WD. 2019 Life on the frontline 2019: a global survey of the working conditions of rangers. Godalming, UK: WWF.

[13] CITES Secretariat. 2019 Report to CITES CoP 18 on Monitoring the Illegal Killing Of Elephants (MIKE). CoP18 Doc. 69.2: 1–20. See https://cites.org/sites/default/files/eng/cop/18/doc/E-CoP18-069-02.pdf.

[14] CITES Secretariat. 2022 Report to the 74th Standing Committee meeting of CITES on the illegal killing of elephants and ivory trade. CITES. See https://cites.org/sites/default/files/eng/com/sc/74/E-SC74-68.pdf (accessed 7 April 2022).

[15] Oyanedel R, Gelcich S, Milner-Gulland EJ. 2020 A synthesis of (non-) compliance theories with applications to small-scale fisheries research and practice. Fish and Fisheries **21**, 1120-1134. (10.1111/faf.12490)

[16] Warchol GL. 2004 The transnational illegal wildlife trade. Crim. Justice Stud. **17**, 57-73. (10.1080/08884310420001679334)

[17] Douglas LR, Alie K. 2014 High-value natural resources: linking wildlife conservation to international conflict, insecurity, and development concerns. Biol. Conserv. **171**, 270-277. (10.1016/j.biocon.2014.01.031)

[18] Titeca K. 2019 Illegal ivory trade as transnational organized crime? An empirical study into ivory traders in Uganda. Br. J. Criminol. **59**, 24-44. (10.1093/bjc/azy009)

[19] Wasser SK et al. 2022 Elephant genotypes reveal the size and connectivity of transnational ivory traffickers. Nat. Hum. Behav. **6**, 371-382. (10.1038/s41562-021-01267-6) 35165434 PMC10693927

[20] Zuur A, Ieno EN, Walker N, Saveliev AA, Smith GM. 2009 Mixed effects models and extensions in ecology with R. New York, NY: Springer.

[21] Burn RW, Underwood FM, Blanc J. 2011 Global trends and factors associated with the illegal killing of elephants: a hierarchical bayesian analysis of carcass encounter data. PLoS ONE **6**, e24165. (10.1371/journal.pone.0024165) 21912670 PMC3166301

[22] Hauenstein S, Kshatriya M, Blanc J, Dormann CF, Beale CM. 2019 African elephant poaching rates correlate with local poverty, national corruption and global ivory price. Nat. Commun. **10**, 1-9. (10.1038/s41467-019-09993-2) 31138804 PMC6538616

[23] Sundberg R, Melander E. 2013 Introducing the UCDP Georeferenced Event Dataset. J. Peace Res. **50**, 523-532. (10.1177/0022343313484347)

[24] Smits J, Steendijk R. 2015 The International Wealth Index (IWI). Soc. Indic. Res. **122**, 65-85. (10.1007/s11205-014-0683-x)

[25] Smits J, Permanyer I. 2019 Data descriptor: the subnational human development database. Sci. Data **6**, 1-15. (10.1038/sdata.2019.38) 30860498 PMC6413757

[26] Weiss DJ et al. 2018 A global map of travel time to cities to assess inequalities in accessibility in 2015. Nature **553**, 333-336. (10.1038/nature25181) 29320477

[27] Do Q-T, Levchenko AA, Ma L, Blanc J, Dublin H, Milliken T. 2021 The price elasticity of African elephant poaching. World Bank Econ. Rev. **35**, 545-562. (10.1093/wber/lhaa008)

[28] Tredennick AT, Hooker G, Ellner SP, Adler PB. 2021 A practical guide to selecting models for exploration, inference, and prediction in ecology. Ecology **102**, e0336. (10.1002/ecy.3336) PMC8187274 33710619

[29] Plummer M. 2003 JAGS: just another Gibbs sampler. See https://sourceforge.net/projects/mcmc-jags.

[30] Su Y-S, Yajima M. 2015 R2jags: using R to Run ‘JAGS’. R package version 0.5-7. See https://rdrr.io/cran/R2jags.

[31] Kéry M, Royle JA. 2020 Applied hierarchical modeling in ecology: analysis of distribution, abundance and species richness in R and BUGS: volume 2: dynamic and advanced models. New York, NY: Academic Press.

[32] van Buuren S, Groothuis-Oudshoorn K. 2011 mice: Multivariate imputation by chained equations in R. J. Stat. Softw. **45**, 1-67.

[33] Roberts DR et al. 2017 Cross-validation strategies for data with temporal, spatial, hierarchical, or phylogenetic structure. Ecography **40**, 913-929. (10.1111/ecog.02881)

[34] Nakagawa S, Schielzeth H. 2013 A general and simple method for obtaining R2 from generalized linear mixed-effects models. Methods Ecol. Evol. **4**, 133-142. (10.1111/j.2041-210x.2012.00261.x)

[35] Gaynor KM, Fiorella KJ, Gregory GH, Kurz DJ, Seto KL, Withey LS, Brashares JS. 2016 War and wildlife: linking armed conflict to conservation. Frontiers in Ecology and the Environment **14**, 533-542. (10.1002/fee.1433) PMC7753333 33362436

[36] Schlossberg S, Gobush KS, Chase MJ, Elkan PW, Grossmann F, Kohi EM. 2020 Understanding the drivers of mortality in African savannah elephants. Ecol. Appl. **30**, 1-15. (10.1002/eap.2131) 32297403

[37] Maisels F et al. 2013 Devastating decline of forest elephants in Central Africa. PLoS ONE **8**, e59469. (10.1371/journal.pone.0059469) 23469289 PMC3587600

[38] Hübschle AM. 2017 The social economy of rhino poaching: of economic freedom fighters, professional hunters and marginalized local people. Curr. Sociol. **65**, 427-447. (10.1177/0011392116673210)

[39] Paudel K, Potter GR, Phelps J. 2020 Conservation enforcement: insights from people incarcerated for wildlife crimes in Nepal. Conserv. Sci. Pract. **2**, 1-11.

[40] Duffy R, St John FAV, Büscher B, Brockington D. 2016 Toward a new understanding of the links between poverty and illegal wildlife hunting. Conserv. Biol. **30**, 14-22. (10.1111/cobi.12622) 26332105 PMC5006885

[41] Wilkie DS, Starkey M, Abernethy K, Effa EN, Telfer P, Godoy R. 2005 Role of prices and wealth in consumer demand for bushmeat in Gabon, Central Africa. Conserv. Biol. **19**, 268-274. (10.1111/j.1523-1739.2005.00372.x)

[42] Jachmann AH, Billiouw M. 1997 Elephant poaching and law enforcement in the Central Luangwa Valley. Zambia **34**, 233-244.

[43] Hilborn R, Arcese P, Borner M, Hando J, Hopcraft G, Loibooki M, Mduma S, Sinclair ARE. 2006 Effective enforcement in a conservation area. Science **314**, 1266. (10.1126/science.1132780) 17124316

[44] Moore JF, Mulindahabi F, Masozera MK, Nichols JD, Hines JE, Turikunkiko E, Oli MK. 2018 Are ranger patrols effective in reducing poaching-related threats within protected areas? J. Appl. Ecol. **55**, 99-107. (10.1111/1365-2664.12965)

[45] Buscaglia E, van Dijk J. 2003 Controlling organized crime and corruption in the public sector. Forum Crime Soc. **3**, 3-34.

[46] Smith RJ, Muir RDJ, Walpole MJ, Balmford A, Leader-Williams N. 2003 Governance and the loss of biodiversity. Nature **426**, 67-70. (10.1038/nature02025) 14603318

[47] Wright SJ, Sanchez-Azofeifa GA, Portillo-Quintero C, Davies D. 2007 Poverty and corruption compromise tropical forest reserves. Ecol. Appl. **17**, 1259-1266. (10.1890/06-1330.1) 17708206

[48] Sundström A. 2016 Corruption and violations of conservation rules: a survey experiment with resource users. World Dev. **85**, 73-83. (10.1016/j.worlddev.2016.04.011)

[49] Bennett EL. 2015 Legal ivory trade in a corrupt world and its impact on African elephant populations. Conserv. Biol. **29**, 54-60. (10.1111/cobi.12377) 25103555

[50] van Uhm DP, Moreto WD. 2018 Corruption within the illegal wildlife trade: a symbiotic and antithetical enterprise. Br. J. Criminol. **58**, 864-885.

[51] Nishihara T. 2012 Demand for forest elephant ivory in Japan. Pachyderm **52**, 55-65.

[52] Wasser SK, Gobush KS. 2019 Conservation: monitoring elephant poaching to prevent a population crash. Curr. Biol. **29**, R627-R630. 10.1016/j.cub.2019.06.009 31386839

[53] Liew JH, Kho ZY, Lim RBH, Dingle C, Bonebrake TC, Sung YH, Dudgeon D. 2021 International socioeconomic inequality drives trade patterns in the global wildlife market. Sci. Adv. **7**, 1-12. 10.1126/sciadv.abf7679 PMC8099177 33952526

[54] Kuiper T. 2022 Replication data for the PIKE 2021 covariate analysis for identifying factors associated with the illegal killing of elephants. Harvard Dataverse, V1 (10.7910/DVN/GNI6DS)

[55] Kuiper T et al. 2023 Drivers and facilitators of the illegal killing of elephants across 64 African sites. Figshare. (10.6084/m9.figshare.c.6350538) PMC9832558 36629103

